# Copolyimide Brushes as a Component of a Hybrid Poly(phenylene Oxide) Membrane for Controlling Gas Separation: Effect of Water, Methanol, and Hexane Vapors

**DOI:** 10.3390/membranes13090805

**Published:** 2023-09-20

**Authors:** Nadezhda Tian, Alexandra Pulyalina, Ilya Faykov, Iosif Gofman, Konstantin Zolotovsky, Galina Polotskaya

**Affiliations:** 1 Institute of Chemistry, Saint Petersburg State University, 198504 Saint Petersburg, Russiapolotskaya@hq.macro.ru (G.P.); 2 Nanomaterial Research Center, Kola Science Center, Russian Academy of Sciences, 184209 Apatity, Russia; 3 Institute of Macromolecular Compounds, Russian Academy of Sciences, 199004 Saint Petersburg, Russia

**Keywords:** poly(*m*-phenylene isophthalamide), mixed matrix membrane, complex modifier, gas separation, vapor saturation

## Abstract

The effect of water, methanol, and hexane vapors on gas permeability was studied in a hybrid membrane containing 5 wt% copolyimide brushes with poly(methyl methacrylate) side chains (PI-g-PMMA) in a poly(phenylene oxide) (PPO) matrix, and in a pristine PPO membrane. These membranes in the form of dense nonporous films were further examined by atomic force microscopy (AFM) and scanning electron microscopy (SEM), as well as by measuring their mechanical and gas transport properties. A gas separation study of the membranes in a dry state and the membranes saturated with water, methanol, and hexane vapors was performed to estimate the effect of each vapor on the H_2_, CO_2_, N_2_ permeability and selectivity in the separation of H_2_/N_2_ and CO_2_/N_2_ pairs. In general, saturation with water, methanol, and hexane vapors caused a decrease in the gas permeability of both membranes. The hybrid membrane containing copolyimide brushes demonstrated enhanced selectivity in the separation of H_2_/N_2_ and CO_2_/N_2_ pairs. It was found that a special effect of the vapors used for membrane saturation is associated with their molar volume. The solubility and diffusion coefficients of N_2_ and CO_2_ were obtained by Grand Canonical Monte Carlo (GCMC) and molecular dynamics (MD) simulations.

## 1. Introduction

Natural gas is the purest type of fossil fuel in terms of ecology [1] since its combustion produces a significantly lower amount of harmful substances compared to other types of fuels. The release of almost 70% of natural gas during its extraction from underground porous rocks is carried out in the process of hydraulic fracturing [2]. In this process, water mixed with various chemicals, including methanol, is injected into the well [3]. The chemicals are added to water to reduce friction and inhibit corrosion. The fluid under high pressure produces hydraulic fracturing, which leads to the release of gas from the rock. When reaching the surface, the gas stream contains 30–70% of the fracturing fluid, containing a mixture of water, chemicals, sand, and other components.

The presence of hard-to-separate components in natural gas limits its use in industry and everyday life [4]. To separate natural gas from complex multicomponent mixtures, the use of membrane technologies is preferable [5], because they are environmentally friendly, energy-saving, and characterized by a simple design [6,7,8,9,10,11]. However, it can be difficult to apply polymer membranes when extracting natural gas on the coastline, since such gas contains a large amount of water. The presence of water vapor affects the mobility of polymer chains, transport channels, and interactions between natural gas and polymer functional groups [12,13,14].

Recently, studies on the effect of liquid vapors (water, methanol, etc.) on the properties of polymer membranes involved in the production and processing of natural gas have been carried out. The most prevalent studies include gas separation using polymer membranes saturated with water vapor [15,16]. A negative effect of humidity on the gas separation characteristics of membranes based on hydrophobic materials has been established [17,18,19]. In the case of hydrophobic polyimides, such as Matrimid [20], a decrease in permeability was observed, which was associated with the competitive sorption and filling of the free volume with water molecules. Scholes et al. [21] reported a decrease in the permeability of CO_2_ and CH_4_ through a polyimide with an increase in relative humidity, whilst the CO_2_/CH_4_ selectivity remained constant in the entire humidity range.

Membranes made of polymeric materials are the most common diffusion membranes for gas separation [22,23,24,25,26,27]. Modification of industrial polymers with various inorganic [28,29], organic [30], and metal–organic frameworks [31,32], as well as polymeric fillers [33,34], represents one of the common approaches to design membranes with improved properties and great prospects. Poly(2,6-dimethyl-1,4-phenylene oxide) (PPO) is a commercially available polymer that is used in gas separation processes and generally demonstrates good permeability but low selectivity, the latter limiting its use [35]. A significant improvement in its gas separation properties was established when modifying PPO with inorganic fillers [36,37,38,39] and fullerene-containing star-shaped macromolecules.

The latest achievement is the development of a hybrid gas separation membrane consisting of PPO modified by the additives of a copolyimide brush having poly(methyl methacrylate) (PI-g-PMMA) side chains [40,41]. The PI-g-PMMA belongs to a new class of graft copolymers, in which the free volume can be controlled by varying the length and density of the side-chain grafting. The introduction of the graft copolyimide with PMMA side chains into the PPO matrix leads to an increase in the membrane selectivity, which indicates the prospects of using this modifier for gas separation membranes.

It was shown in [40,41] that membranes containing 5 wt% PI-g-PMMA demonstrate an optimal tradeoff between permeability and selectivity in the separation of H_2_/N_2_ and CO_2_/N_2_ mixtures. Studies of copolyimide brushes with PMMA side chains of different lengths (m = 50 or 150 units) showed that the introduction of the grafted copolyimide PI-g-PMMA with a shorter chain into the PPO matrix provides the best effect and allows one to obtain a membrane with an increased selectivity.

Therefore, the object of this work is to evaluate the effect of water, methanol, and hexane vapors on the permeability of H_2_, CO_2_, and N_2,_ and determine the gas separation properties of a hybrid membrane containing 5 wt% PI-g-PMMA copolyimide brushes in the PPO matrix. To assess the peculiarities of membrane saturation with water, methanol, and hexane vapors with respect to the transport of small gas molecules, comparative studies of the hybrid PPO/PI-g-PMMA and pristine PPO membranes were carried out.

## 2. Materials and Methods

### 2.1. Materials

**Figure 1:**
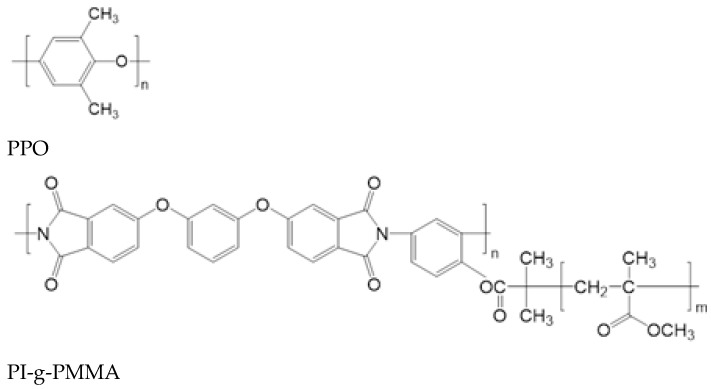
Chemical structures of PPO and PI-g-PMMA.

Commercial powdered PPO (Figure 1) with a molecular weight (MW) of 338 × 10^3^ g/mol and density of 1.054 g/cm^3^ was purchased from Sigma-Aldrich Chemie GmbH (Schnelldorf, Germany). Macromolecular brushes with a polyimide (PI) backbone and poly(methyl methacrylate) (PMMA) side chains (PI-g-PMMA) were synthesized by polymerization of methyl methacrylate (MMA) using a multifunctional PI as a macroinitiator (*M*_n_ = 35.2∙10^3^, *M*_w_/*M*_n_ = 2.06), as described in [42]. The grafting density of the PMMA side chains was 80%, and the average degree of PMMA polymerization (*m*) was 65 ÷ 78.

### 2.2. Membrane Preparation

The hybrid membrane studied in this work was prepared according to the technique described in [40,41]. In particular, PPO/PI-g-PMMA compositions containing 5 wt% PI-g-PMMA were obtained by mixing individual solutions of 3 wt% PPO and 3 wt% PI-g-PMMA in chloroform, with thorough stirring, on a magnetic stirrer for 2 h at 40 °C, followed by degassing. The resulting PPO/PI-g-PMMA casting solution represented a transparent single-phase system due to the high affinity of each of the polymer components to the solvent. Dense non-porous PPO and PPO/PI-g-PMMA membranes having a thickness of ~80–90 µm were prepared by casting a solution of the polymer mixture onto a cellophane surface. The said solution was then subjected to evaporation of the solvent and drying in a vacuum at 40 °C. Then, the membrane was separated from the cellophane and dried in a vacuum at 40 °C to a constant weight. The resulting PPO/PI-g-PMMA membranes lost their transparency, which indicates phase segregation occurred between the polymer components. Any evidence of aggregation and phase separation was observed by AFM and SEM, as discussed below.

### 2.3. Computer Simulation

To simulate the sorption and diffusion processes of N_2_ and CO_2_ molecules in PPO, a cell model of the PPO membrane was designed using the Materials Studio 7.0 (MS) software platform. The COMPASS II force field [43] was used for building the cell and subsequent calculations. Furthermore, calculations of the free volume and its space distribution in the polymer were carried out. The solubility and diffusion coefficients of N_2_ and CO_2_ were obtained by analyzing an average number of gas molecules and the dynamic behavior of the molecules in the cell by Grand Canonical Monte Carlo (GCMC) and molecular dynamics (MD) simulations, respectively. The Metropolis method [44] was applied to successfully generate a chain of configurations, whilst the Ewald summation method [45] was used to calculate the electrostatic interactions. The equations of motion were solved by the leapfrog algorithm [46]. Finally, the effect of water molecules on the dynamic and thermodynamic characteristics of gases was studied. The modeling process is described in more detail in the “Supplementary Materials” Files.

### 2.4. Membrane Characterization

Three-dimensional images AFM images of the membrane surfaces were obtained using a Nanoscope III apparatus (Digital Instruments, Santa Barbara, CA, USA) equipped with a Digital Instruments 1553D scanner under the following measurement conditions: tapping mode and OTESPA silicon cantilevers (Veeco Instruments, Dourdan, France) with a radius of 5 nm and an oscillation frequency of 300 kHz.

Cross-section SEM images of the membranes were obtained by using a JSM-35 electron microscope (JEOL Ltd., Tokyo, Japan). Before carrying out the experiment, the cross-sections were coated with a layer of gold (20 nm) by cathodic sputtering using a Quorum 150 sputter coater (Quorum, Laughton, UK).

The mechanical characteristics of the films, such as Young’s modulus, *E*, the break stress, *σ_b_*, and the ultimate deformation, *ε_b_*, were determined using an AG-100kNX Plus setup (Shimadzu, Kyoto, Japan) operating in a uniaxial extension mode. Strip-like samples with a width of 2 mm and a length of 30 mm were stretched at room temperature at a rate of 10 mm/min, as required by ASTM D638.

The film density, *ρ*, was estimated using the flotation method with a laboratory-made measurement unit. The sucrose aqueous solution was used to equilibrate the specimens at 25 °C. Samples of 0.05–0.10 g were used; the error of measurements was ± 0.0001 g/cm^3^.

The Hildebrand solubility parameters, *δ*, of the polymers were calculated as a ratio of the cohesion energy, *E_coh_*, and the molar volume, *V_m_*, of individual groups constituting the polymer molecules. The following equation was applied [47]:

(1) $$\delta_{i}={\left(\frac{\sum E_{coh,\;i}}{\sum V_{m,\;i}}\right)}^{1/2}$$

### 2.5. Gas Transport

**Figure 2:**
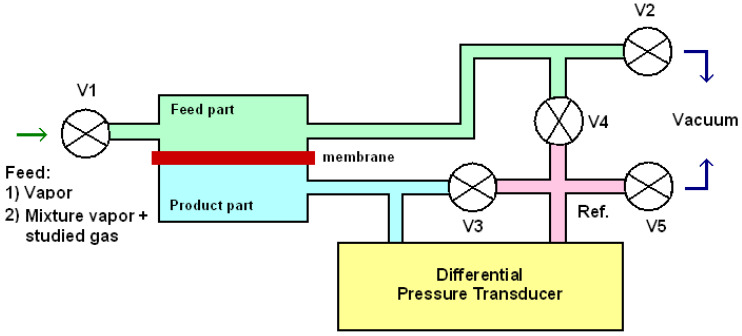
Scheme of laboratory gas separation apparatus for measurements in a swollen state.

The transport properties of the membranes were studied by measuring the permeability of H_2_, N_2_, and CO_2_ gases through the membranes saturated with water, methanol, or hexane vapors at 30 °C. A laboratory high-vacuum apparatus with a static permeation cell and an effective membrane area of 5.25 cm^2^ was used for the measurements (Figure 2). Before the experiment, a membrane sample was placed in a vapor atmosphere under a certain pressure of 30 kPa for the time period of 48 h required to saturate the sample (using valve V1, with valves V3 and V4 being also opened). At the beginning of the permeation experiment, the whole apparatus was filled with vapor, and the pressure in the product part was maintained constant. A mixture of the studied gas and vapor under a constant pressure, *p_i_*, of 150 kPa was added to the feed part through valve V1. A differential pressure transducer was used to measure the pressure increase in the calibrated volume. A reference volume (see, Ref. on Figure 2) was kept under the initial vapor pressure, with valves V3, V4, and V5 closed during the steady state.

The permeability, *P*, was determined from the pressure increase, Δ*p_p_*, in a calibrated volume, *V_p_*, of the product part of the cell per the time interval, Δt, during the steady state permeation (as registered by a pressure transducer). The following equation was used to calculate the permeability [40]:

(2) $$P=\frac{∆p_{p}}{∆t}\cdot \frac{V_{p}\cdot l}{S\cdot p_{i}}\cdot \frac{1}{RT},$$

where *l* is the membrane thickness, *S* is the membrane area, *T* is temperature, and *R* is the gas constant. All the measurements were carried out at 30 °C.

The ideal selectivity (*α_i/j_*) for gas *i* with respect to gas *j* was calculated according to the following equation:

(3) $$\alpha_{i/j}=\frac{P_{i}}{P_{j}},$$

## 3. Results and Discussions

### 3.1. Membrane Structure

Two dense nonporous membranes based on pristine PPO and hybrid PPO/PI-g-PMMA containing 5 wt% copolyimide brushes were prepared by solvent evaporation. First of all, the general difference in structure of the hybrid PPO/PI-g-PMMA membrane from the pristine PPO was estimated by AFM and SEM.

**Figure 3:**
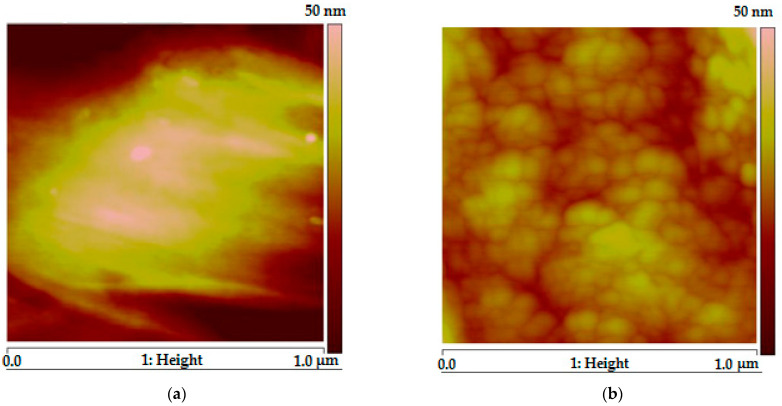
AFM images of PPO (**a**) and PPO/PI-g-PMMA (**b**) membrane surfaces.

Figure 3 shows AFM images of the pristine PPO and PPO/PI-g-PMMA membrane surfaces. The surface of the PPO membrane is relatively smooth. The addition of the PI-g-PMMA modifier leads to a complication of the structure, namely, the formation of spherical fragments on the membrane surface. This feature indicates the aggregation of macromolecules when mixing the components of the hybrid membrane.

**Figure 4:**
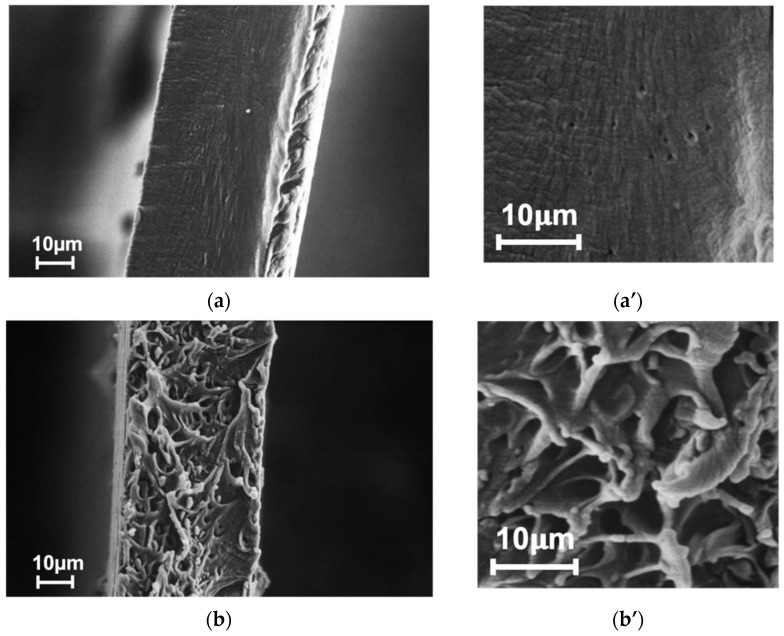
SEM micrographs of cross-sections of (**a**,**a′**) PPO and (**b**,**b′**) PPO/PI-g-PMMA membranes.

Figure 4 shows SEM micrographs of the pristine PPO and hybrid PPO/PI-g-PMMA membrane cross-sections. The cross-section of the PPO (Figure 4a) contains small light lines, which can be attributed to the crystalline phase of PPO [41]. Figure 4b illustrates the phase separation that occurs in the case of the hybrid membrane: there are clearly defined areas ranging in size from one to several microns against the background of a large number of light strands, which are probably formed during the destruction of the partially crystalline matrix. The areas within the strands probably represent an isolated PI-g-PMMA phase. The presence of a cluster of modifying molecules in the PPO matrix with clearly defined phase boundaries can affect the mechanical and transport characteristics of the hybrid membrane.

### 3.2. Mechanical Properties

Table 1 shows the mechanical properties of the pristine PPO and hybrid PPO/PI-g-PMMA membranes. The characteristics of the material determined during the mechanical tests are as follows: Young’s modulus (*E*), tensile strength (*σ_b_*), and ultimate strain (*ε_b_*). It was established that the mechanical strength parameters (Young’s modulus and tensile strength) remained almost at the same level as the corresponding parameters for the pristine PPO films. At the same time, the film elasticity (the ultimate strain) decreases after the inclusion of 5 wt% PI-g-PMMA in the PPO matrix. This fact can be associated with a change in the degree of crystallinity of the partially crystalline PPO after the introduction of the copolyimide brushes, as has been shown in [41]. The reduction in the ultimate strain of the hybrid membrane could arise from the phase-separated structure observed in Figure 4. The overall level of mechanical properties is quite satisfactory for the membrane operating conditions during gas separation [48].

Previously, it was shown that the density of films made from PI-g-PMMA ranged from 1.18 to 1.34 g cm^−3^, depending on the length of the side chains and the density of their grafting [49]. Since the PPO films have a lower density (1.054 g cm^−3^), the introduction of a filler with a higher density into the PPO leads to the formation of hybrid membranes with higher density compared to pristine PPO (Table 1).

**Figure 5:**
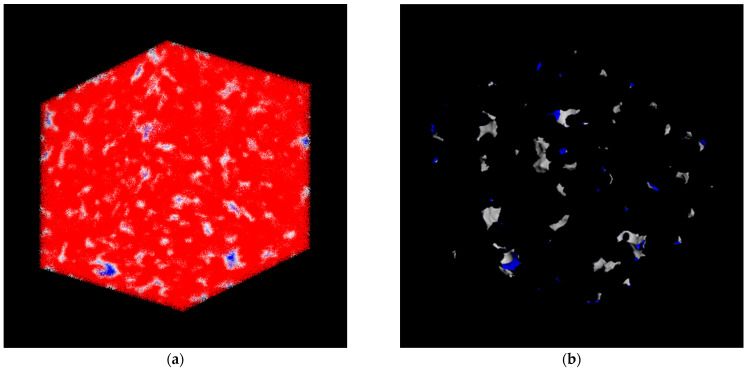
The visualization of the distribution of free volume in the PPO matrix: (**a**) atomic volume field and (**b**) accessible solvent surface.

A cell model of the PPO membrane was designed using the MS software platform, and further calculations of the free volume and its space distribution in the polymer were carried out. Figure 5a,b shows the visualization of the distribution of free volume in the PPO matrix and the complex structure of channels and cavities of the polymer.

As seen in Figure 5a, the atomic volume field contains values of some distance function, such that the isosurfaces of that field characterize the geometry and interaction of an atomistic structure polymer with gases. The red regions relate to the areas with high potential energy (non-accessible for gas molecules), while the blue regions represent negative energy (favorable for insert). The white layer corresponds to the area of possible contact of gas molecules with the polymer matrix.

Figure 5b demonstrates the accessible solvent surface (d = 0.3 nm). This surface represents a locus of the probe center as the probe rolls over the van der Waals surface. In other words, the said surface describes the space that could be occupied over externally accessible regions.

### 3.3. Transport Properties

**Figure 6:**
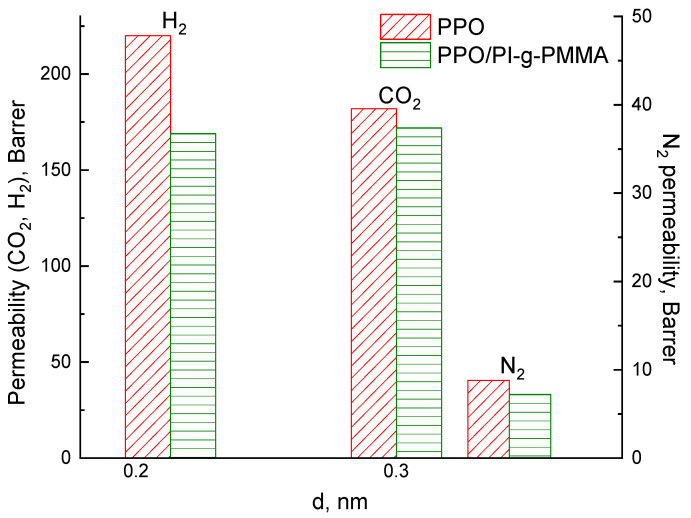
Dependence of H_2_, N_2_, and CO_2_ permeability on effective diameter (d) of gas molecules for PPO and PPO/PI-g-PMMA membranes saturated with water vapor.

In this work, the permeability of H_2_, N_2_, and CO_2_ gases was estimated under saturation of PPO and PPO/PI-g-PMMA membranes with water, methanol, and hexane vapors (Figure 6). First of all, the effect of the PI-g-PMMA inclusion in the PPO matrix was studied for the membranes saturated with water vapor. Figure 6 shows the dependence of gas permeability on the effective diameter of gas molecules in the PPO and PPO/PI-g-PMMA membranes saturated with water vapor. In particular, it was established that the gas permeability decreases in the following order: H_2_ (0.210 nm) > CO_2_ (0.302 nm) > N_2_ (0.304 nm), which is associated with an increase in the effective diameter of the gas molecules. The introduction of the PI-g-PMMA brush in the PPO membrane reduces the permeability of all the studied gases.

The CO_2_ permeability through both membranes is high, but special features occur, as discussed elsewhere [50]. The difference in CO_2_ permeability through the PPO/PI-g-PMMA compared to the PPO membrane is much lower than the difference in H_2_ or N_2_ permeability through these membranes. This phenomenon is due to the specific interaction of CO_2_ with PPO and especially PMMA macromolecules, which provide facilitated transport of this gas species [51].

A comprehensive study on the gas permeability of the membranes saturated with vapors of water, methanol, and especially hexane is carried out for the first time. Thus, such an analysis requires data on the physical properties of the substances used to saturate the membranes, which are presented in Table 2. Attention should be drawn to the significant difference in the molar volumes of the above substances. The molar volume of hexane significantly exceeds that of water and methanol. Table 2 also shows the solubility parameters (*δ*) of water, methanol, and hexane, which can be used to evaluate the interaction of these liquids with the polymer. According to the theory of solubility [52], the smaller the difference between the solubility parameters of a polymer and a low molecular weight substance, |Δ*δ*|, the greater their affinity. The solubility parameter, δ, is 18.2 MPa^1/2^ for pure PPO, 22.5 MPa^1/2^ for PI, and 19.0 MPa^1/2^ for PMMA. The latter means that the components of the polymer membrane will have the best affinity to methanol (|Δ*δ*| < 11.5) and hexane (|Δ*δ*| < 4.1) and the poorest affinity to water (|Δ*δ*| < 31.4). However, the smaller size of water molecules provides a significant advantage in terms of the penetration rate through polymer membranes.

**Figure 7:**
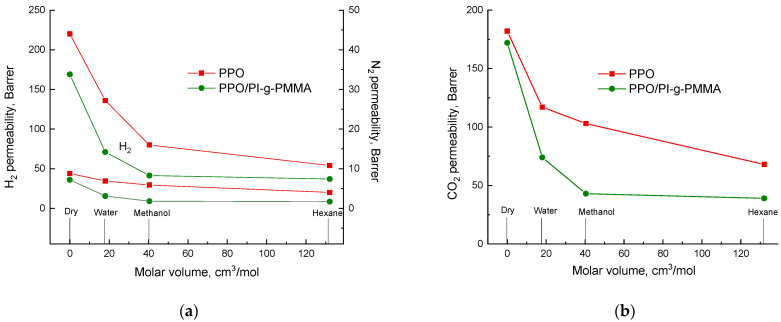
Dependence of the permeability coefficients for (**a**) H_2_, N_2_, and (**b**) CO_2_ on the molar volume of liquids whose vapors saturate PPO and PPO/PI-g-PMMA membranes.

Figure 7a,b shows the permeability of H_2_, N_2_, and CO_2_ through the vapor-saturated PPO and PPO/PI-g-PMMA membranes, depending on the molar volume of the vapor species. The molar volume of vapors used to saturate the membranes increases in the following order: water < methanol < hexane. As expected, saturation with water vapor, methanol, and hexane caused a decrease in the gas permeability of both membranes [53]. For all the gases, an increase in the molar volume of the vapors leads to a decrease in the membrane permeability. Moreover, the difference between the permeability of dry membranes and vapor-saturated membranes is quite significant. Such behavior (a decrease in permeability) is due to the fact that water, methanol, and hexane molecules fill the elements of the free volume and reduce its availability for the diffusion of non-condensable penetrants H_2_, N_2_, and CO_2_.

According to the modern theory of permeability [54], both factors (*D* and *S*) that determine the permeability coefficient (*P* = *D·S*) are considered in the analysis of gas transport. Along with the diffusion coefficient (*D*) as a kinetic component of gas transport, the solubility coefficient (*S*) as a thermodynamic factor should be taken into account. The kinetic parameter of gas transport depends on the physical features of the PPO/PI-g-PMMA membrane having a phase-separated structure. This structure is characterized by an increased density on the phase boundaries and a decreased free volume. The solubility coefficient depends on the gas nature, as well as the nature of the membrane material. As can be seen from Figure 6, in the case of the hybrid membrane containing PI-g-PMMA brushes with functional hydrophilic fragments in the form of grafted PMMA chains, a decrease in the gas permeability becomes greater than for the hydrophobic PPO membrane.

Using the MD and GCMC simulations, we determined the diffusion and solubility coefficients of CO_2_ and N_2_ gases in a dry PPO membrane and a PPO membrane saturated with water vapors (Table 3). The values obtained support the complexity of changes that occur when the membrane is saturated with water vapors. A change in the local field reduces the solubility of the components while filling the membrane cavities with water prevents the diffusion of molecules. Moreover, due to a decrease in the free volume of the adsorbate–adsorbate interaction, they begin to strongly affect the diffusion.

As a result of the modeling, the main contributions to a decrease in the permeability of CO_2_ and N_2_ in the PPO saturated with water vapor were identified. Based on the data obtained, the following conclusions on the effects of membrane saturation on gas transport can be made:

- The channels and cavities of the polymer saturated with water vapor become more polar, which leads to a local increase in the dielectric constant of the membrane and a decrease in the nitrogen solubility.
- Filling the cavities with H_2_O molecules reduces the available volume required for the relay nature of gas diffusion in the polymer; therefore, the standard deviation of the adsorbate molecules from the local equilibrium positions decreases. This change especially affects the CO_2_ molecules due to their size and strong electrostatic effects with water.

**Figure 8:**
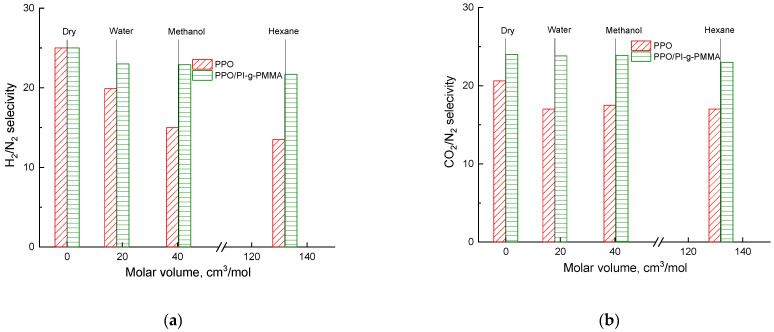
Dependence of selectivity in separation of gas pairs (**a**) H_2_/N_2_ and (**b**) CO_2_/N_2_ on the molar volume of liquids whose vapors saturate PPO and PPO/PI-g-PMMA membranes.

Figure 8 shows a change in the H_2_/N_2_ and CO_2_/N_2_ selectivity of the PPO and PPO/PI-g-PMMA membranes saturated with water, methanol, and hexane vapors. The total level of selectivity of the hybrid PPO/PI-g-PMMA membrane significantly exceeds the selectivity of the PPO membrane. Furthermore, the selective properties of the PPO membrane decrease upon saturation with water vapors and vapors of organic substances. At the same time, the selectivity of the hybrid PPO/PI-g-PMMA membrane retains its elevated level, even in the saturated state.

We would like to highlight the excellent stability of properties of both membranes during long-term gas separation experiments in a saturated state and the reproducibility of the results obtained.

## 4. Conclusions

The novelty of this work consists in studying the effects of three vapors on the gas transport properties of saturated polymer membranes. This problem occurs when the extraction of natural gas from underground porous rocks is carried out in the process of hydraulic fracturing.

The objects of this study are dense nonporous PPO and PPO/PI-g-PMMA membranes prepared by solvent evaporation. Structural differences in the hybrid membrane containing 5 wt% copolyimide brushes from the pristine PPO membrane were shown by AFM and SEM. The presence of PI-g-PMMA in the hybrid membrane has a substantial effect on its mechanical and gas transport properties. In particular, the PI-g-PMMA filler decreases the permeability of H_2_, N_2_, and CO_2_ molecules through the membrane matrix and leads to enhancement of the membrane selectivity. Saturation of both membranes with water, methanol, and hexane vapors allows one to estimate the influence of each vapor on the H_2_, CO_2_, N_2,_ permeability, and selectivity in the separation of H_2_/N_2_ and CO_2_/N_2_ pairs. In general, saturation with water, methanol, and hexane vapors caused a decrease in the gas permeability of both types of membranes. Saturation with water vapor, and even more so with vapors of organic substances, reduces the selective properties of the pristine PPO membrane. At the same time, the selectivity of the hybrid PPO/PI-g-PMMA membrane retains its higher level. It was found that the specific effect of vapors used to saturate the membranes is associated with their molar volume, which increases in the following order: water < methanol < hexane. Special attention is drawn to the excellent stability of the properties of both membranes during long-term gas separation experiments in a saturated state.

## Figures and Tables

**Table 1:** Mechanical properties of membranes.

Membrane	E, GPa	*σ_b_*, MPa	*ε_b_*, %	Density, g/cm^3^
PPO	1.90 ± 0.16	44 ± 1	14 ± 2	1.060 ± 0.060
PPO/PI-g-PMMA	1.87 ± 0.20	48 ± 2	5.7 ± 0.3	1.065 ± 0.060

**Table 2:** Physical properties of liquids.

Liquid	Mol. Weight, g/mol	Density, g/cm^3^	Mol. Volume, cm^3^/mol	Dynamic Viscosity, mPa∙s	Solubility Parameter, *δ*, MPa^1/2^
Water	18.0	0.997	18.0	1.0	49.6
Methanol	32.0	0.792	40.4	0.55	29.7
Hexane	86.2	0.655	131.6	0.30	14.9

**Table 3:** Gas transport parameters of PPO membrane.

Gas	Diffusion Coefficient, D_298_, 10^−6^ cm^2^·s^−1^	Solubility Coefficient, S_298_, 10^−4^ mol·dm^−3^·bar^−1^	Simulation Permeability Coefficient, P, Barrer	Experimental Permeability Coefficient, P, Barrer
N_2_	7.15 ± 0.10	0.40 ± 0.10	9.6	8.8
N_2_ (for membrane saturated with water vapor)	6.10 ± 0.16	0.23 ± 0.05	4.7	6.9
CO_2_	3.93 ± 0.12	14.90 ± 0.16	196	182
CO_2_ (for membrane saturated with water vapor)	2.92 ± 0.11	13.6 ± 0.13	133	117

## Data Availability

The data presented in this study are available on request from the corresponding author.

## Supplementary Materials

The following supporting information can be downloaded at: https://www.mdpi.com/article/10.3390/membranes13090805/s1, Figure S1: Sketch Toolbar—creation of 2,6-Xylenol, CO_2_, N_2_ (with centroid), H_2_O molecules; Figure S2: Building Homopolymer—creation of an oligomer from 30 monomer units of 2,6-Xylenol; Figure S3: Amorphous Cell Construction—creation of a cell of 30 chains (density 1.057 g/cm^3^); Figure S4: (a) Results of GCMC calculations and (b) Mean square displacement of gas molecules; Table S1: Forcite Geometry Optimization—molecular optimization; Table S2: Forcite Geometry Optimization—chain optimization; Table S3: Forcite Geometry Optimization—cell optimization; Table S4: Forcite Anneal—MD relaxing the chain; Table S5: Forcite Dynamics—MD relaxing the cell; Table S6: Sorption Fixed Pressure—GCMC saturation with water vapor; Table S7: Sorption Fixed Pressure—GCMC predicting the loading (with and without water); Table S8: Forcite Dynamics—MD calculating the mean square displacement molecules (with and without water).

## Abbreviations

The abbreviations and symbols used in this manuscript are as follows:

AFM	Atomic force microscopy
GCMC	Grand Canonical Monte-Carlo
MD	Molecular dynamics
MMA	Methyl methacrylate
MS	Materials Studio
MW	Molecular weight
PI	Polyimide
PMMA	Poly(methyl methacrylate)
PI-g-PMMA	Copolyimide brush with poly(methyl methacrylate) side chains
PPO	Poly(2,6-dimethyl-1,4-phenylene oxide)
SEM	Scanning electron microscopy
*E*	Young’s modulus
*α_i/j_*	ideal selectivity
*σ_b_*	break stress
Σ*_b_*	ultimate deformation
*ρ*	density
*δ*	Hildebrand solubility parameter
*D*	diffusion coefficient
*E_coh_*	cohesion energy
*V_m_*	molar volume
*P*	permeability
*V_p_*	calibrated volume of the product part of the cell
*t*	time
*l*	membrane thickness
*S*	membrane area/solubility coefficient
*T*	temperature
*R*	gas constant
