# Supplementary material for: Copolyimide Brushes as a Component of a Hybrid Poly(phenylene Oxide) Membrane for Controlling Gas Separation: Effect of Water, Methanol, and Hexane Vapors
Source: Membranes (Basel). 2023 Sep 20;13(9):805. doi: 10.3390/membranes13090805 (PMC10536049; doi:10.3390/membranes13090805)
Supplement: Supplementary file 1 [file membranes-13-00805-s001.zip › membranes-2569888-supplementary.pdf]

membranes

Supplementary Materials

Copolyimide Brushes as a Component of a Hybrid Membrane for Controlling Gas Separation: Effect of Water, Methanol and Hexane Vapors

Nadezhda Tian [1], Alexandra Pulyalina [1,2,*], Ilya Faykov [1], Iosif Gofman [3], Konstantin Zolotovsky [1] and Galina Polotskaya [1,3]

[1] Institute of Chemistry, Saint Petersburg State University, Saint Petersburg 198504, Russia; polotskaya@hq.macro.ru (G.P.)
[2] Nanomaterial Research Center, Kola Science Centre, Russian Academy of Sciences, Apatity 184209, Russia
[3] Institute of Macromolecular Compounds, Russian Academy of Sciences, Saint Petersburg 199004, Russia
* Correspondence: a.pulyalina@spbu.ru; Tel.: +78-124-284-805

In this work, we followed the specific calculation algorithm as follows:

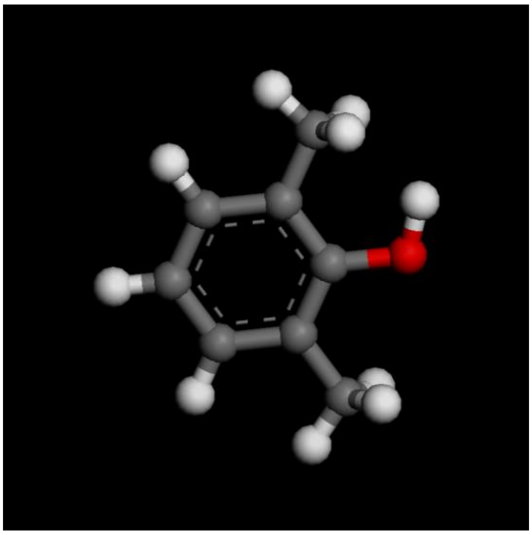

2,6-Xylenol

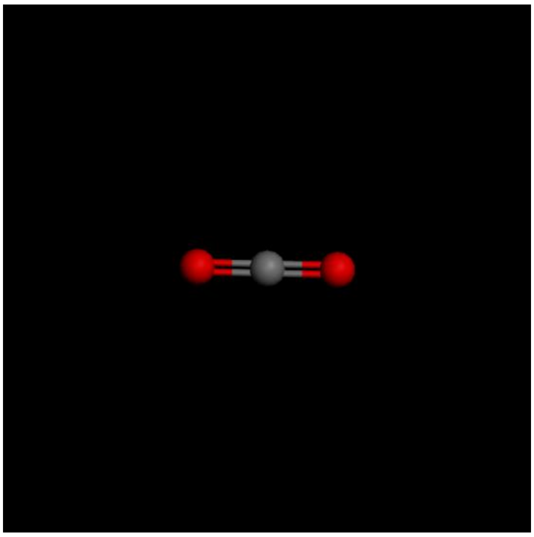

CO_2

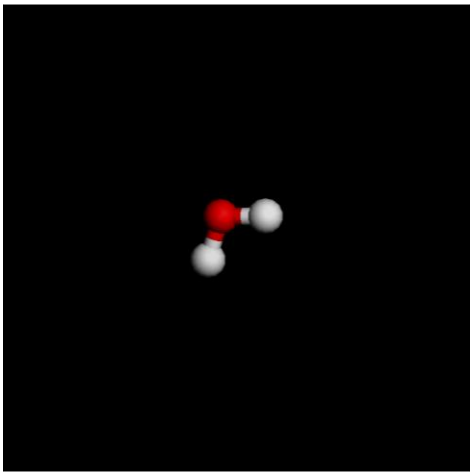

H_2O

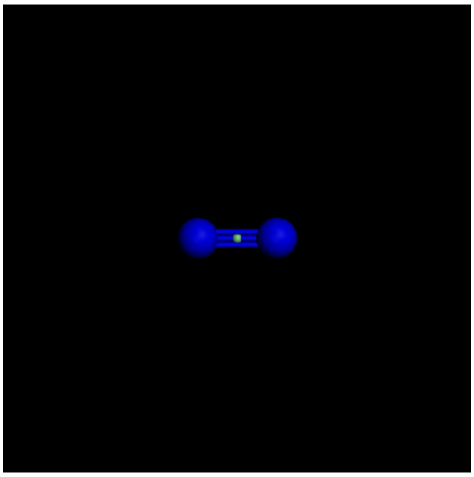

N_2 (with centroid)

Figure S1. Sketch Toolbar — creation of 2,6-Xylenol, CO_2, N_2 (with centroid), H_2O molecules.

Table S1. Forcite Geometry Optimization — molecular optimization.

**Geometry optimization parameters**		**Energy parameters**			
Algorithm	Smart	Forcefield		COMPASSII (Version 1.1)	
Convergence tolerance		Charges		Forcefield assigned	
Energy	2e-005 kcal/mol	Electrostatic terms		van der Waals terms	
Force	0.001 kcal/mol/Å	Summation method	Atom based	Summation method	Atom based
Displacement	1e-005 Å	Truncation method	Cubic spline	Truncation method	Cubic spline
Maximum number of iterations	500	Cutoff distance	18.5 Å	Cutoff distance	18.5 Å
Motion groups rigid	NO	Spline width	1 Å	Spline width	1 Å
		Buffer width	0.5 Å	Buffer width	0.5 Å

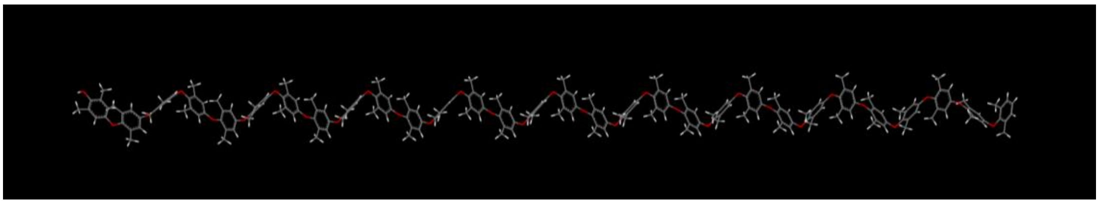

Figure S2. Building Homopolymer — creation of an oligomer from 30 monomer units of 2,6-Xylenol.

Table S2. Forcite Geometry Optimization — chain optimization.

**Geometry optimization parameters**	**Energy parameters**

Algorithm	Smart	Forcefield		COMPASSII (Version 1.1)	
Convergence tolerance		Charges		Forcefield assigned	
Energy	2e-005 kcal/mol	Electrostatic terms		van der Waals terms	
Force	0.001 kcal/mol/Å	Summation method	Atom based	Summation method	Atom based
Displacement	1e-005 Å	Truncation method	Cubic spline	Truncation method	Cubic spline
Maximum number of iterations	500	Cutoff distance	18.5 Å	Cutoff distance	18.5 Å
Motion groups rigid	NO	Spline width	1 Å	Spline width	1 Å
		Buffer width	0.5 Å	Buffer width	0.5 Å

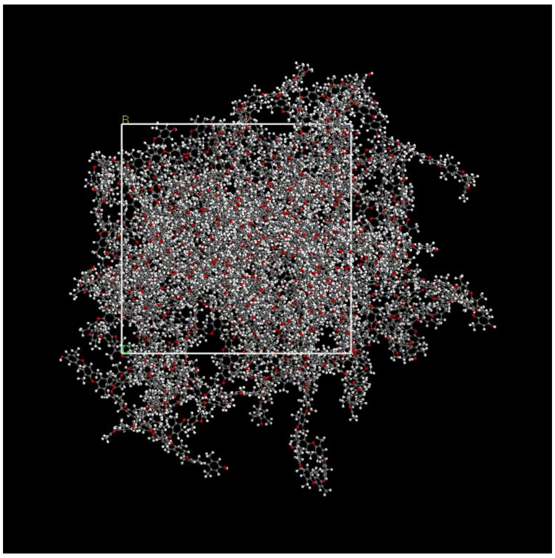

“origin” visualization

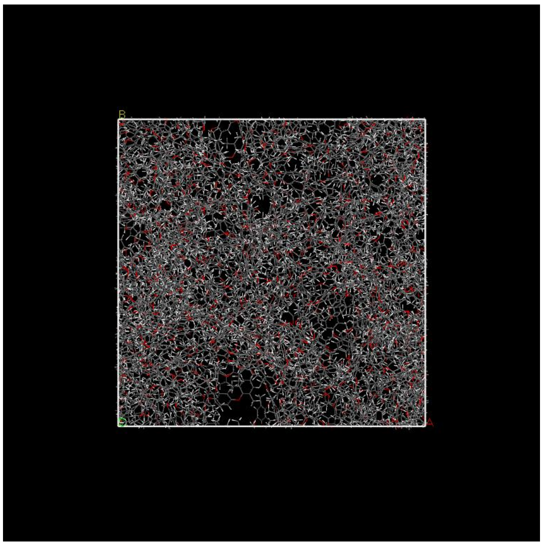

“in-Cell” stick visualization

Figure S3. Amorphous Cell Construction — creation of a cell of 30 chains (density 1.057 g/cm^3).

Table S3. Forcite Geometry Optimization — cell optimization.

**Geometry optimization parameters**		**Energy parameters**			
Algorithm	Smart	Forcefield		COMPASSII (Version 1.1)	
Convergence tolerance		Charges		Forcefield assigned	
Energy	2e-005 kcal/mol	Electrostatic terms		van der Waals terms	
Force	0.001 kcal/mol/A	Summation method	Ewald	Summation method	Atom based

Maximum number of iterations	500	Accuracy	0.001 kcal/mol	Truncation method	Cubic spline
External pressure	0 GPa	Buffer width	0.5 A	Cutoff distance	18.5 A
Motion groups rigid	NO			Spline width	1 A
Optimize cell	NO			Long range correction	YES
				Buffer width	0.5 A

Table S4. Forcite Anneal – MD relaxing the chain.

**Anneal parameters**		**Dynamics parameters**	
Number of anneal cycles	5	Ensemble	NVT
Initial temperature	300.00 K	Control method	NHL
Mid-cycle temperature	500.00 K	Q ratio	0.0100000
Heating ramps per cycle	5	Decay constant	1.0000000 ps
Dynamics steps per ramp	100	Timestep	1.00 fs
Total number of dynamics steps	5000	Initial velocities	Random
Optimize annealed structure	False		

Table S5. Forcite Dynamics – MD relaxing the cell.

**Dynamics parameters**		**Energy parameters**	
Ensemble	NPT	Forcefield	COMPASSII (Version 1.1)
Temperature	298.00 K	Charges	Forcefield assigned
Control method	NHL	Electrostatic terms	
Q ratio	0.0100000	Summation method	Ewald
Decay constant	1.0000000 ps	Accuracy	0.0001 kcal/mol
Pressure	0,000101325 GPa	Buffer width	0.5 A
Control method	Parrinello	van der Waals terms	
Cell time constant	1.0000000 ps	Summation method	Atom based
Timestep	1.00 fs	Truncation method	Cubic spline
Number of steps	1000000	Cutoff distance	15.5 A
Duration	1000 ps	Spline width	1 A
Initial velocities	Random	Long range correction	YES
		Buffer width	0.5 A

Table S6. Sorption Fixed pressure – GCMC saturation with water vapor.

**Fixed pressure calculation parameters**		**Metropolis Monte Carlo method parameters**			**Fixed pressure calculation**	
Monte Carlo method	Metropolis	Parameter	Ratio	Maximum amplitude	Sorbate	Fugacity, kPa
Equilibration steps	100000	Exchange	2.000	-	H_2O	3.000
Production steps	8000000	Rotate	1.000	5 deg		
Sample interval	25 steps	Translate	1.000	1 A		
		Regrowth	0.100	-		

Table S7. Sorption Fixed pressure – GCMC predicting the loading (with and without water).

**Fixed pressure calculation parameters**		**Metropolis Monte Carlo method parameters**			**Fixed pressure calculation**	
Monte Carlo method	Metropolis	Parameter	Ratio	Maximum amplitude	Sorbate	Fugacity, kPa
Equilibration steps	100000	Exchange	2.000	-	CO_2	100.000
Production steps	8000000	Rotate	1.000	5 deg	N_2	100.000
Sample interval	25 steps	Translate	1.000	1 A		
Monte Carlo method	Metropolis	Regrowth	0.100	-		

Table S8. Forcite Dynamics – MD calculating the mean square displacement molecules (with and without water).

**Dynamics parameters**		**Energy parameters**	
Ensemble	NPT	Forcefield	COMPASSII (Version 1.1)
Temperature	298.00 K	Charges	Forcefield assigned
Control method	NHL	Electrostatic terms	
Q ratio	0.0100000	Summation method	Ewald
Decay constant	1.0000000 ps	Accuracy	0.0001 kcal/mol
Pressure	0,0002 GPa	Buffer width	0.5 A
Control method	Parrinello	van der Waals terms	
Cell time constant	1.0000000 ps	Summation method	Atom based
Timestep	1.00 fs	Truncation method	Cubic spline
Number of steps	1000000	Cutoff distance	15.5 A
Duration	1000 ps	Spline width	1 A
Initial velocities	Random	Long range correction	YES
		Buffer width	0.5 A

The gas solubility in the polymer was calculated by simulating GCMC at a fixed pressure and determining the average number of gas molecules in the material. This allows to calculate the gas solubility coefficient as:

$$S_i = \frac{\langle N_i \rangle}{N_A p_i V} \quad \textbf{(S1)}$$

The gas diffusivity in the polymer was calculated by running a molecular dynamics simulation and determining the mean square displacement of the gas in the material. This allows you to calculate the self-diffusivity coefficient of the gas and gives an insight into the overall diffusivity. The mean square displacement of the particles with respect to their original position is obtained as the second moment of their distribution at t > 0, and is related to the diffusion coefficient as follows:

$$D_i = \frac{1}{6N_i} \lim_{t\to\infty} \left(\frac{d\langle [r_i(t) - r_i(0)]^2 \rangle}{dt} \right) \quad \text{(S2)}$$

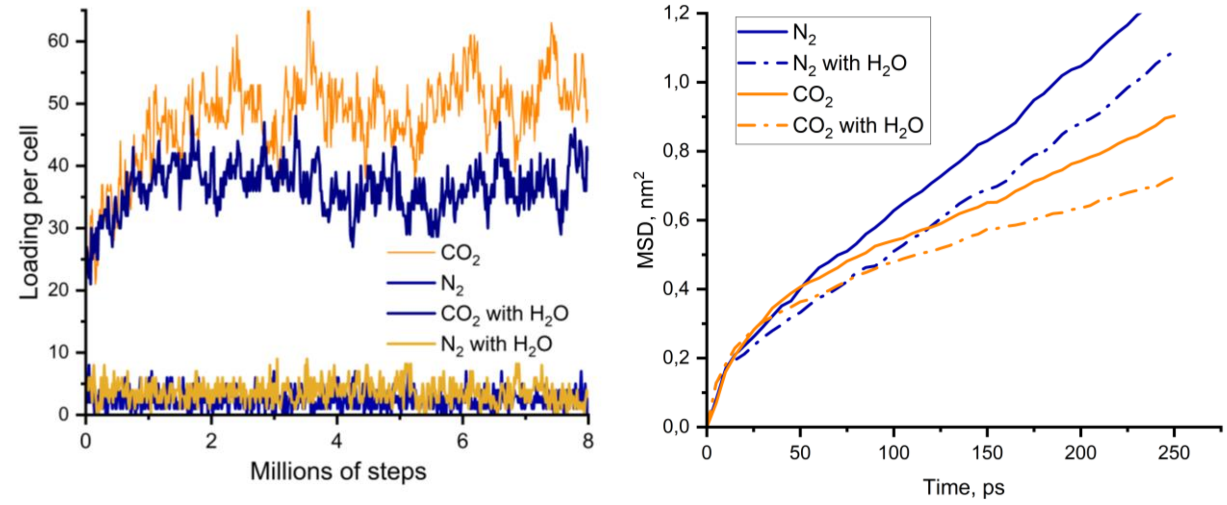

(**a**) (**b**)

Figure S4. (**a**) Results of GCMC calculations and (**b**) Mean square displacement of gas molecules.
